# Effects of the COVID-19 pandemic on the mental health of medical students and young physicians in Germany: Gender-specific results of an online survey

**DOI:** 10.1016/j.heliyon.2023.e23727

**Published:** 2023-12-19

**Authors:** Marie Halfmann, Noah Castioni, Lea Wetzel, Anne Koopmann, Sarah König, Astrid Schmieder

**Affiliations:** a Department of Addictive Behavior and Addiction Medicine, Central Institute of Mental Health (CIMH), Mannheim, Germany; b Feuerlein Centre on Translational Addiction Medicine (FCTS), University of Heidelberg, Germany; c Institute for Medical Teaching and Training Research, University Hospital Wuerzburg, Germany; d Clinic for Dermatology, Venereology and Allergology, University Hospital Wuerzburg, Germany

**Keywords:** Mental health, Covid-19, Medical students, Sex differences

## Abstract

**Background:**

Healthcare workers and medical students faced new challenges during the COVID-19 pandemic. Processes within many hospitals were completely disrupted. In addition, the face to face teaching of medical students was drastically reduced. Those at risk of developing mental health problems appear to be younger health care workers and women.

**Objective:**

To investigate potential COVID-19 pandemic-related gender differences in psychological distress among medical students and physicians in their first years of practice.

**Design and setting:**

An anonymous survey was carried out online between December 1, 2021, and March 31, 2022, at the Mannheim Medical Faculty and the Würzburg Medical Faculty, Germany, after obtaining informed consent. Primary outcome measures were changes in anxiety and depression symptoms using the Hospital Anxiety and Depression Scale (HADS), and changes in participants' current quality of life using the WHO Quality of Life BREF.

**Results:**

The results show wave-like courses for perceived anxiety and burden overlapping with the course of the COVID-19 incidence. In comparison to men, women showed a significant higher increase in HADS (*p* = *0.005*) and a reduced life quality (*p* = *0.007*) after COVID-19. Both sexes showed different frequencies of the factors influencing quality of life, with the presence of a previous mental illness and mean anxiety having a significant higher negative impact in women.

**Conclusion:**

Future and young female physicians reported a disproportionate higher burden during COVID-19 compared to their male colleges. These observations suggest an increased need for support and prevention efforts especially in this vulnerable population.

## 1 Introduction

770 million infections and 6.96 million deaths worldwide were caused by the SARS-CoV-2 pandemic between December 2019 and the end of September 2023 [1]. Comprehensive measures were established in most parts of the world to contain the contagions, with often far-reaching restrictions on social life, as well as university and work settings. Both the health care system as a whole and the medical education of students and young physicians have also been challenged by university closures, widespread conversion to online teaching formats, changing working conditions, and increased risk of SARS-CoV-2 exposure in the work environment [2,3]. Fear of one's own potential infection or transmitting COVID-19 to relatives and varying availability of protective equipment at work or study sites, as well as changing personal and professional circumstances, are potential stressors and place prospective and practicing physicians in a vulnerable position [4].

The impact of SARS-CoV-2 infection on physical health has been the focus of several studies, with concurrent consideration of potential consequences for psychological well-being. A scoping review of 34 studies revealed a range of symptoms including fatigue, arthralgia, pain, and reduced physical capacity, as well as depression, and anxiety after COVID-19 in the general population. Here, the women surveyed showed higher scores concerning anxiety and depression [5].

Prior to the COVID-19 pandemic, doctors were already a highly mentally burdened group. Before the outbreak of the COVID-19 pandemic, the aggregated prevalence of depression or depressed symptoms among doctors was 28.8% [6] compared to 9.2% in the general population [7] with considerable in-between-study variability, which is potentially due to variations in survey techniques and questionnaires. Female physicians appear to be at a greater risk for suicide when compared to the general female population [8].

Various meta-analyses of studies conducted during the pandemic on the prevalence of mental distress among healthcare workers (HCW) yielded pooled prevalence scores for anxiety of 25.8% among physicians [9] and 23.2% among HCW [10], and pooled prevalence scores for depression of 20.5% in physicians [9], and 22.8% [10], or 24% in HCW [11]. Another review described high prevalence rates of anxiety and depression among surveyed HCW, with several studies showing that women had more symptoms of anxiety as well as higher severity for depressive symptoms [12].

German medical students showed significant increases in depressive symptoms and loneliness during the COVID-19 pandemic [13], as well as greater study-related worries early on [14]. Risk factors for higher stress levels and a decreased well-being were female gender [[15], [16], [17]], a subjectively lower social status [16], and a younger age [18]. Also among HCW, in addition to female gender and inadequate protection against COVID-19 infection, younger age is a risk factor for anxiety and depression [12,19]. Therefore, young female physicians in their first years of practice and female medical students are expected to be particularly vulnerable.

It is therefore to be feared that the two and a half years of daily study and work under the changed conditions and potential burdens may have adverse effects on the mental health of medical students and physicians, particularly for women in their early years of practicing medicine. To assess how the COVID-19 pandemic differed in affecting the mental health of women and men, this anonymous online survey asked both medical students and young physicians of different specialties about their life quality during the COVID-19 pandemic, in order to identify potential risk groups and stressors and to evaluate the need for support and prevention measures at the faculties and workplaces.

## 2 Material and methods

### 2.1 Study population

The information was obtained by administering a confidential online questionnaire to participants at the Mannheim Medical Faculty of the University of Heidelberg and the Wuerzburg Medical Faculty during the period of December 01, 2021, to March 31, 2022. Medical studies in Germany are carried out in six-year terms and consist of preclinical and clinical phases, which culminate in a practical year. In this study, all medical students as well as young physicians already in practice for up to 10 years after completing medical studies were eligible to participate in the survey. This enabled participants up to 45 years of age to take part in the study.

Participants were recruited through the student deans' offices and student councils at the respective universities, as well as calls for participants from the various hospitals and secretariats of the different specialties via email and public relations. All survey participants provided informed consent prior to their participation.

The survey was conducted utilizing SoSci.surveys software (version 3.2.40-im SoSci Survey GmbH, Munich, Germany).

The study received approval from the Ethics Committee II of the Mannheim Medical Faculty at the University of Heidelberg (file number: 2021-645), as well as the Ethics Committee of the University of Wuerzburg (file number 2021-120901). Additionally, the study was registered with the German Registry for Clinical Studies (DRKS-ID: DRKS00028984).

For the subject to be examined, the data of both students and physicians were taken into account, resulting in an analysis of the entire data set (N = 668). For further research, respondents were also divided into subgroups by sex, women (N = 484) and men (N = 184). Due to a small subsample (N = 3) compared to the other sexes, individuals with diverse gender were excluded in this analysis.

### 2.2 Survey procedure

A total of 668 participants were included in the survey. The survey consisted of validated questionnaires and self-generated queries. Participants were asked socio-demographic questions regarding their age, gender, family income, and family status. Moreover, they provided feedback on the availability of protective equipment at their workplace or university (rated on a 5-point scale ranging from “not at all sufficient” to “completely sufficient”), as well as their perceived level of threat in regard to the COVID-19 pandemic on individual, national, and global levels (rated as “low,” “medium,” or “high”). Additionally, participants reported their levels of anxiety and burden caused by the pandemic, as well as the impact on their family, social, and professional lives (rated as “positive,” “negative,” or “neutral”).

Participants retrospectively assessed their subjective anxiety, burden, and impairment during seven distinct time periods throughout the pandemic from spring 2020 to fall 2021. They utilized categorical questions with either 3 or 5 level choices. Furthermore, they were asked about any pre-existing psychiatric condition with the options of “yes” or “no”. The study assessed changes in anxiety and depression symptoms using the well-validated German version of the Hospital Anxiety and Depression Scale (HADS) [20], along with the WHO Quality of Life BREF (WHOQOL BREF) [21]. Current quality of life was evaluated with reliability values ranging between α = 0.57 and α = 0.88.

### 2.3 Statistical analysis

IBM SPSS version 27 (IBM Corporation, Armonk, NY, USA) [22] was utilized for statistical calculations, employing a 2-sided significance level of α = 0.05 for all tests. Frequency distributions were presented as absolute case numbers and percentage frequencies for the entire sample and two subgroups (women vs. men).

The non-parametric Friedman test determined variations in personally perceived anxiety and burden from spring 2020 to fall 2021. The total and subsample scores of HADS-A/D before (bo) and after (ao) pandemic onset were analyzed through paired-sample T-tests. For testing significant differences, independent samples t-tests were used to compare life quality mean scores (WHOQOL BREF) between women and men.

To assess the impact of variables such as age, pre-pandemic mental illness, protective equipment availability, mean anxiety, mean burden, and changes in HADS sum score on the quality of life of both students and physicians, a multifactorial ANOVA was performed. Additionally, we conducted further multifactorial ANOVA analyses to determine the variables that affect participants' quality of life across the five domains of the WHO QOL BREF. Specifically, we performed separate ANOVAs for male and female subgroups.

## 3 Results

### 3.1 Sample description

Complete data from 668 students and physicians were included in the analysis. The sample primarily consisted of females (n = 484, 72.5%) with ages ranging from 18 to 42 years.

Table 1 presents a summary of sociodemographic data and COVID-19 specific inquiries for the whole study population and gender subgroups.

**Table 1:** Sociodemographic and COVID-19 data.

		Total sample		Women		Men	
		N	%	N	%	N	%
		668	100	484	72.5	184	27.5
Approbation^a^							
	Yes	107	16.0	66	13.6	41	22.3
	No	561	84.0	418	86.4	143	77.7
Family status							
	Married	49	7.3	33	6.8	16	8.7
	Single/Single living	393	58.8	280	57.9	113	61.4
	Liaised/enganged	61	9.1	51	10.5	10	5.4
	Living with partner	135	20.2	93	19.2	42	22.8
	Living separately	9	1.3	7	1.4	2	1.1
	Divorced	1	0.1	1	0.2	0	0
	Others	20	3.0	19	3.9	1	0.5
Socioeconomic status							
	Low	16	2.4	15	3.1	1	0.5
	Insufficient	20	3.0	14	2.9	6	3.3
	Medium	178	26.6	135	27.9	43	23.4
	Sufficient	290	43.4	205	42.4	85	46.2
	High	164	24.6	115	23.8	49	26.6
Age in years (M, SD)^a^		24.40 (4.75)		24.00 (4.50)		25.45 (5.23)	
Protection equipment (M, SD)		4.10 (0.94)		4.06 (0.95)		4.21 (0.89)	
Prior mental illness							
	Yes	77	11.5	61	12.6	16	8.7
	No	591	88.5	423	87.4	168	91.3
Psychosocial support							
	Student organization	19	2.8	9	1.9	10	5.4
	Self-help programme	7	1.0	6	1.2	1	0.5
	Supervision	7	1.0	3	0.6	4	2.2
	Psychotherapy	97	14.5	74	15.3	23	12.5
	None	519	77.7	377	77.9	142	77.2
	Others	19	2.8	15	3.1	4	2.2

Participants with incomplete data were excluded from the study.

The evaluation of socioeconomic status reflects a subjective perception without an objective foundation.

The adequacy of the protection equipment was rated on a 5-point Likert scale ranging from 1, indicating not sufficient, to 5, indicating fully sufficient.

N: sample size; M: mean; SD: standard deviation.

a Significant differences between the groups.

### 3.2 Subjectively perceived anxiety

**Fig. 1:**
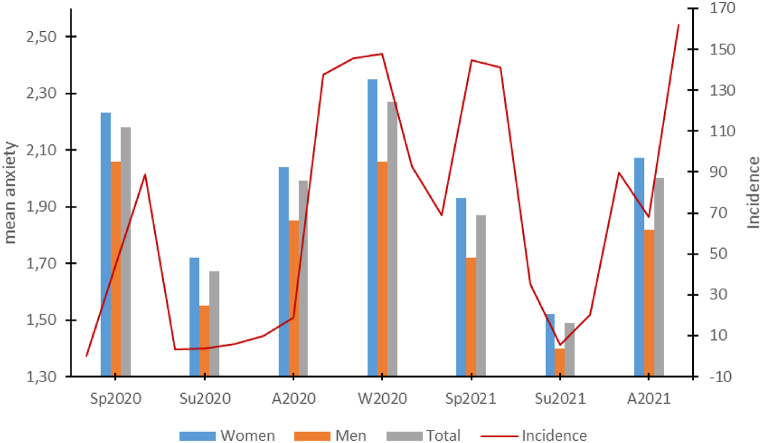
Time course of subjective anxiety. Legend: Average anxiety scores measured at various time points for the entire study population and subgroups of women and men. Data is related to the nationwide COVID-19 seven-day incidence rates over time. Source material is from Robert-Koch-Institut (RKI) and can be found at https://www.rki.de/DE/Content/InfAZ/N/Neuartiges_Coronavirus/Situationsberichte/COVID-19-Trends/COVID-19-Trends.html?__blob=publicationFile#/home; Access: May 02, 2022 [23].

Between the seven measurement times, differences in the subjects’ subjectively perceived anxiety for the entire sample (Friedman test: Chi^2^(6) = 827.31, *p* < *0.001*, n = 668) as well as the subsamples of women (Chi^2^(6) = 633.06, *p* < *0.001*, n = 484) and men (Chi^2^(6) = 198.87, *p* < *0.001*, n = 184) (see Fig. 1) were detected.

Overlapping with the COVID-19 incidence pattern, anxiety scores showed a wave-like pattern, increasing in the fall, winter, and spring and decreasing in the summer. For a comprehensive analysis, please refer to the online supplement, which includes a complete table of post-hoc Dunn-Bonferroni tests comparing each measurement time point.

### 3.3 Subjectively perceived burden

**Fig. 2:**
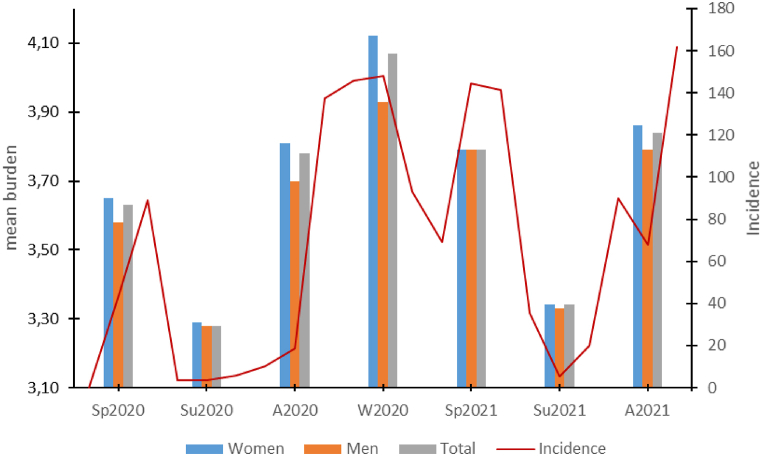
Time course of subjective burden. Legend: Mean burden at different time points for the entire study population and subsamples of women and men, relative to nationwide COVID-19 7-day incidence scores. Source: Robert-Koch-Institut (RKI): COVID-19 Trends in Germany: https://www.rki.de/DE/Content/InfAZ/N/Neuartiges_Coronavirus/Situationsberichte/COVID-19-Trends/COVID-19-Trends.html?__blob=publicationFile#/home; Access: May 02, 2022) [23].

A wave-like pattern in subjective perception of burden was observed. The Friedman test demonstrated a significant difference between all seven time points of measurement, including the sample as a whole (Chi2(6) = 565.11, p < 0.001, n = 668), as well as the subsamples of women (Chi2(6) = 446.78, p < 0.001, n = 484) and men (Chi2(6) = 124.35, p < 0.001, n = 184) (see Fig. 2).

### 3.4 Depression score progression over time

**Fig. 3:**
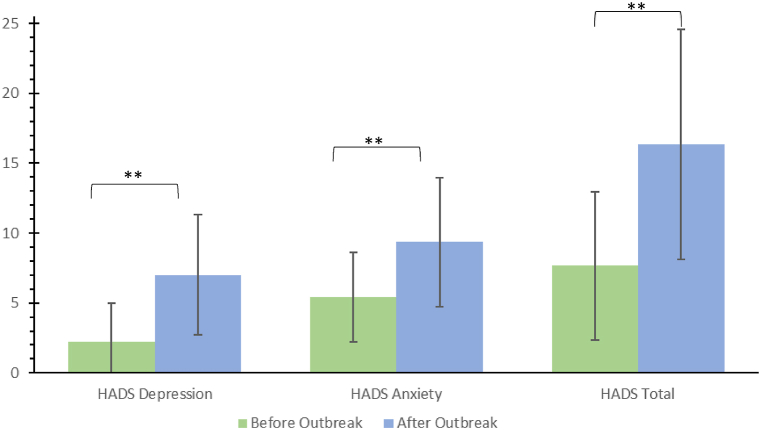
Changes of the Hospital Anxiety and Depression Scale (HADS) pre- versus post COVID-19 outbreak. Legend: Change in sum scores on the HADS total scale and its subscales anxiety and depression pre- and post-outbreak of the pandemic in March 2020.

Mean scores for depression and anxiety on the HADS scale significantly increased in the total sample comparing scores before (bo: M = 7.66, SD = 5.29) to after (ao: M = 16.36, SD = 8.21) the onset of the COVID pandemic (t(667) = −32.29, p.001). A subscale analysis of the HADS scale showed a significant increase from before (bo: M = 2.25, SD = 2.73) to after (ao: M = 5.41, SD = 4.31) the onset of the pandemic for the depression subscale (HADS-D) (t(667) = −32.18, p.001) as well as for the anxiety subscale (HADS-A) from before (bo: M = 5.41, SD = 3.22) to after (ao: M = 9.36, SD = 4.60) the onset of the pandemic (t(667) = −27.74, p < 0.001). It is noteworthy that only 8.8% of all participants met the cut-off of 15 for a clinically significant value prior to the onset of the pandemic, while 49.1% met the cut-off after the onset. The depression and anxiety subscales showed a similar pattern with a cut-off of 8 for a clinically evident value (depression: before onset - 4.2% ≥ 8, after onset - 31.1% ≥ 8; anxiety: before onset - 15.7% ≥ 8, after onset - 54.2% ≥ 8, refer to Fig. 3 for details).

### 3.5 Difference in change in depression scores according to gender

**Fig. 4:**
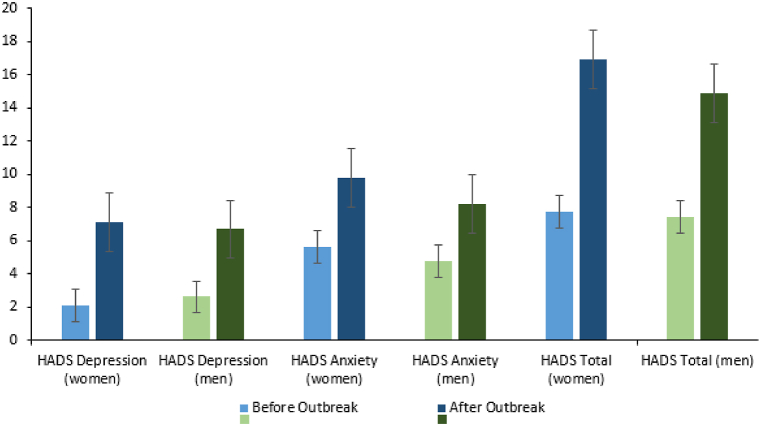
Changes in HADS sum scores in sex comparison. Legend: Changes in HADS sum scores for the HADS total scale, anxiety subscale, and depression subscale before the outbreak of the pandemic and after the outbreak in March 2020 for both women and men.

Male participants displayed a comparatively smaller escalation in their depression scores after the pandemic outbreak in contrast to the female participants. This divergence was statistically significant for the HADS full scale (m: M = 7.48, SD = 7.29; w: M = 9.17, SD = 6.79, t(666) = 2.802, p = 0.005), along with its anxiety (m: M = 3.41, SD = 3.86; w: M = 4.15, SD = 3.56, t(666) = 2.312, p = 0.021) and depression (m: M = 4.07, SD = 3.97; w: M = 5.02, SD = 3.73, t(666) = 2.880, p = 0.004) subscales (refer to Fig. 4).

### 3.6 Effects of the pandemic on quality of life

Table 2 shows the participants' subjective assessment of quality of life across different dimensions of the WHOQOL BREF, including the total sample, men and women two years after the pandemic onset.

**Table 2:** Quality of life according to WHOQOL BREF after 2 years of pandemic.

*Descriptive statistics*							
	Total sample		Women		Men		
	M	SD	M	SD	M	SD	
WHO Domain 1: Global	63.94	22.03	63.48	21.36	65.15	23.72	
WHO Domain 2: Physical	73.34	16.32	72.81	16.17	74.75	16.68	
WHO Domain 3: Psychological	61.68	19.83	60.41^a^	19.39	65.01^a^	20.61	
WHO Domain 4: Social relationships	62.69	22.91	64.03^a^	23.15	59.15^a^	21.94	
WHO Domain 5: Environmental	74.35	14.13	74.47	14.12	74.01	14.22	

M: mean; SD: standard deviation.

a Significant difference between women and men p < 0.005.

In the domain social relationships, female participants scored significantly higher than male participants (t(666) = 2.471, p = 0.014, |d| = 0.214). Conversely, in the psychological quality of life domain, male participants scores significantly higher than female participants (t(666) = −2.695, p = 0.007, |d| = 0.233). Among the subsamples for overall quality of life (t(302.44) = −0.834, p = 0.405), physical quality of life (t(666) = −1.373, p = 0.170), and environmental quality of life (t(666) = 0.372, p = 0.710) there was no significant difference.

### 3.7 Factors influencing quality of life during the pandemic

The presence of a prior mental illness (F(1,658) = 18.184, p < 0.001, ηp2 = 0.027), differences in depression scores before and after the onset of the pandemic (F(1,658) = 109. 825, p < 0.001, ηp2 = 0.143), the mean burden (F(1,658) = 8.693, p = 0.003, ηp2 = 0.013), and the presence of infection protection equipment (F(1,658) = 4.048, p = 0.045, ηp2 = 0.006) significantly impacted the global quality of life.

Lower life quality scores were found to have a correlation with a history of mental illness (B = −9.993, t(666) = −4.264, p < 0.001), as well as higher burden scores (B = −4.602, t(666) = −2.948, p = 0.003) and greater differences in depression scores (B = −1.258, t(666) = −10.480, p < 0.001). Additionally, less adequate protection equipment against infection was linked to worsened quality of life (B = 1.622, t(666) = 2.012, p < 0.045).

Sex (*p* = 0*.*267), age (*p* = 0*.*668), working with COVID-19 patients vs. not dealing with them (*p* = 0*.*505), and mean anxiety (*p* = 0*.*251) did not significantly correlate with overall quality of life.

### 3.8 Factors influencing the WHO QOL BREF domains by sex during the pandemic

In order to determine the individual factors influencing the different domains of quality of life of the WHO QOL BREF for the two subgroups women vs. men, multi-factorial ANOVAS were calculated separately for the sexes and the domains (Table 3). Differences in the frequency of the influencing factors, as well as different distributions in the different domains between the two sexes were revealed. This was also evident in the two domains of the WHOQOL BREF showing a significant difference in the quality of life between the women and men surveyed (psychological and social relationships).

**Table 3:** Factors influencing participants’ quality of life by sex.

	Women			Men		
	F	Significance	Partial eta-squared η_p_^2^	F	Significance	Partial eta-squared η_p_^2^
WHO Domain 1: Global						
Previous mental illness	17,905	**,000**	,036	3,120	,079	,018
Mean anxiety	,691	,406	,001	,475	,492	,003
Mean burden	4,855	**,028**	,010	4,002	**,047**	,022
Difference HADS sum score (bo/ao)	102,197	**,000**	,177	14,918	**,000**	,079
Age	1,417	,234	,003	,601	,439	,003
Infection protection equipment	1,428	,233	,003	3,297	,071	,018
WHO Domain 2: Physical						
Previous mental illness	27,888	**,000**	,055	3,069	,082	,017
Mean anxiety	6,162	**,013**	,013	,013	,908	,000
Mean burden	6,475	**,011**	,013	15,456	**,000**	,081
Difference HADS sum score (bo/ao)	69,099	**,000**	,127	17,821	**,000**	,092
Age	3,622	,058	,008	1,245	,266	,007
Infection protection equipment	2,445	,119	,005	1,506	,221	,009
WHO Domain 3: Psychological						
Previous mental illness	38,732	**,000**	,075	4,529	**,035**	,025
Mean anxiety	4,133	**,043**	,009	,615	,434	,004
Mean burden	4,239	**,040**	,009	5,796	**,017**	,032
Difference HADS sum score (bo/ao)	109,968	**,000**	,188	29,278	**,000**	,143
Age	5,265	**,022**	,011	,001	,971	,000
Infection protection equipment	1,036	,309	,002	4,668	**,032**	,026
WHO Domain 4: Social relationships						
Previous mental illness	18,130	**,000**	,037	6,062	**,015**	,033
Mean anxiety	2,976	,085	,006	,426	,515	,002
Mean burden	,379	,539	,001	2,802	,096	,016
Difference HADS sum score (bo/ao)	44,079	**,000**	,085	12,586	**,000**	,067
Age	,511	,475	,001	7,129	**,008**	,039
Infection protection equipment	4,675	**,031**	,010	3,413	,066	,019
WHO Domain 5: Environmental						
Previous mental illness	3,214	,074	,007	1,153	,284	,007
Mean anxiety	5,982	**,015**	,012	2,818	,095	,016
Mean burden	15,242	**,000**	,031	16,026	**,000**	,084
Difference HADS sum score (bo/ao)	38,678	**,000**	,075	3,538	,062	,020
Age	,372	,542	,001	,859	,355	,005
Infection protection equipment	18,604	**,000**	,038	2,308	,130	,013

Bold highlighted numbers mark significant influencing factors.

The multifactorial ANOVA for the psychological domain showed that for both women and men, the presence of a prior mental illness (w: F(1,476) = 38.732, *p* < 0*.*001, ηp^2^ = 0.075; m: F(1,175) = 4.529, *p* = 0*.*035, ηp^2^ = 0.025), mean burden (w: F(1,476) = 4.239*, p* = 0*.*040, ηp^2^ = 0.009; m: F(1,175) = 5.796, *p* = 0*.*017, ηp^2^ = 0.032) and the difference in depression scores before and after the onset of the pandemic (w: F(1,476) = 109.968, *p* < 0*.*001, ηp^2^ = 0.188; m: F(1,175) = 29.278, *p* < 0*.*001, ηp^2^ = 0.143) were relevant factors for the quality of life. In addition, for women, higher anxiety scores (F(1,476) = 4,133, *p* = 0*.*043, ηp^2^ = 0.009; B = −3.805, *t*(483) = -2.033, *p* = 0*.*043) and lower age (F(1,476) = 5,265, *p* = 0*.*022, ηp^2^ = 0.011; B = 0.377, *t*(483) = 2.295, *p* = 0*.*022) resulted in lower psychological quality of life scores. In contrast, the men's analysis of variance revealed that the presence of infection protection equipment (F(1,175) = 4.668, *p* = *0.032*, ηp^2^ = 0.026) significantly affected men's psychological quality of life, with infection protection equipment rated as insufficient (B = 3.280, t(182) = 2.160, p = 0.032), resulting in lower values.

Regarding social relationships, a previous psychiatric disease (w: F(1,476) = 18.130, p < 0.001, ηp2 = 0.037; m: F(1,175) = 6.062, p = 0.015, ηp2 = 0.033) and the difference in depression scores before and after the pandemic (w: F(1,476) = 44.079, p < 0.001, ηp2 = 0.085; m: F(1,175) = 12.586, p < 0.001, ηp2 = 0.067) in both subgroups significantly reduced quality of life. In addition, for women, infection protection equipment subjectively rated as less adequate (F(1,476) = 4,675, *p* = 0*.*031, ηp^2^ = 0.010; B = 2.211, *t*(483) = 2.162, *p* = 0*.*031) led to lower social quality of life scores. Among men, lower age (F(1,175) = 7,129, *p* = 0.008, ηp^2^ = 0.0.39; B = 0.788, *t*(182) = 2.670, *p* = 0*.*008) was associated with lower social quality of life scores.

## 4 Discussion

The evolution of the HADS over the course of the pandemic shows different dynamics according to sex. It is known, that there was a pre-existing “sex gap” in depression and anxiety symptoms already prior to the outbreak of the pandemic, though this is surely not the only explanation for the sex differences assessed in our study [24]. A complicating factor may be that female study participants reported lower average perceived COVID-19 protective equipment than male study participants, which may result in additional stress. In addition, women have a lower psychological quality of life as measured by the WHO QOL-BREF. In studies of the general population, other factors that negatively influenced anxiety and depression in women during the COVID-19 pandemic were higher stress levels and more worries about the virus, both health-related and financial. In addition, women were more likely to worry about possible COVID-19 infection in family members or loved ones [25]. The significantly higher QOL domain social relationships scores among female participants fits with previous findings that women tend to have social networks with fewer but stronger ties [26].

Overlapping with the German COVID-19 incidences, our survey revealed periodic fluctuations in self-reported anxiety and personal burden, with higher levels of both during the colder seasons, particularly among women. The increase of values in the measurement time point “spring 2020” has also been observed in other publications among HCW [10,27] and coincides with the generally increased subjectively perceived anxiety and stress caused by COVID-19 in the German general population [28]. It should be noted that for the measurement time point “spring 2021,” i.e., a good year after the outbreak of the pandemic, a decrease in perceived anxiety compared with the previous measurement time point “winter 2020” can be observed, although the COVID-19 incidence in spring reaches similar peak values as in winter 2020 [23]. This can partly be explained by the approval of three vaccines for COVID-19 in December/January 2021 by the European Medicines Agency. In the spring of 2021, a large proportion of medical staff, including residents and young doctors, had the opportunity to be vaccinated against COVID-19, which could have led to a reduction in anxiety. In general, we found a significant increase in depression and anxiety scores after the pandemic outbreak. According to the results of the HADS items, about half of the subjects showed a clinically conspicuous depression score in the course of the pandemic. This contrasts with previous international reviews in which just over 20% of subjects had clinically salient depression scores [10,29], as well as studies in the German general population in which only 23% of women and 21% of men had clinically salient HADS scores [30]. Here, the different origin of the data is probably of significance. For example, the primary studies were mainly conducted in Asia or outside Europe. Finally, it is important to consider the age structure of this survey, which, with an average age of 24.4 years, is significantly younger than the age structure in the German population [31]. Psychological distress from the COVID-19 pandemic was empirically shown to be greater in younger individuals [28]. This is in accordance with our here presented study data.

In our study, predictors of lower quality of life were pre-existing mental illness, higher difference in depression scores, higher burden scores, and inadequate COVID-19 protective equipment. Here, the presence of infection protection equipment is consistent with findings from others, as in both the past MERS and SARS epidemics and the COVID-19 pandemic, direct work with affected patients and the circumstances of that work are relevant to susceptibility to depressive and anxiety symptoms [32]. Looking at the factors influencing quality of life in each WHOQOL BREF domain in a sex comparison, it seems striking that the presence of a previous mental illness tends to be a risk factor on quality of life more often in women than in the men (w: 4/5 domains, m: 2/5). Furthermore, mean anxiety did not prove to be a significant influencing factor in any of the domains for the male respondents, whereas it had a significant main effect on quality of life in three of the five domains for the female respondents. The reason for this can only be speculated. It may be that women experience more anxiety than men, regardless of the pandemic [33]. Furthermore, the gender care gap could have an impact on this. For example, women in Germany perform on average 52% [34] more care work, such as child rearing, nursing or housework, than men. The largest gap, with a difference of more than 2 h of daily work, can be observed in age groups in their early and mid-30s, which made up a notable proportion of the participants in this study. Evidence suggests that this preexisting disparity was exacerbated by the Covid 19 pandemic. For example, women were more likely to have to forego employment to provide care work than men [35]. Measures that explicitly focus on the situation of working women, such as more flexibility in their employment could provide significant assistance to a vulnerable group and help to decrease extra-professional duties.

The study has several limitations. The most important confounding factor is the retrospective nature of the survey. There is no guarantee that participants can accurately recall how they felt at seven earlier points in time. In addition, the survey was conducted over a period of several months, so the circumstances under which the survey was completed could vary among study participants. In particular, the omicron variant of SARS-CoV-2, which became widespread in late 2021, may have influenced study participants’ perceptions and therefore a bias can be supposed. Furthermore, the survey was only conducted at two medical faculties. Therefore, data cannot be extrapolated to all young medical students and physicians in Germany and elsewhere, as there were significant differences in COVID-19 incidence and preventive measures in different German states and medical schools. Another limitation of this study is the analysis of data from only two genders, male and female, owing to a small number of individuals with diverse gender (N = 3) and the statistical exclusion of these individuals. Future research should include all genders if sufficient case numbers can ensure statistical accuracy. In our study, we found that the female sex was overrepresented (72.5%) compared to the gender distribution of all medical students in Germany (65.13% female; 34.87% male; [36]) and practicing physicians (49.9% women among all physicians and psychotherapists in 2021) [37].

In summary, the survey data revealed an enormous pattern of stress among trainees and young medical staff due to and during the COVID-19 pandemic. Furthermore, clear differences, which probably go beyond the pre-existing “sex gap”, were observed between the female and male study participants. Therefore, the COVID-19 pandemic not only perpetuated the sex gap between men and women in terms of depression and anxiety symptoms, but also seemed to exacerbate it. In addition, our data confirmed that medical personnel are more psychologically stressed than the general population. Based on our data, support and prevention measures seem to be important, especially for vulnerable groups such as young medical students and physicians with a special focus on women.

## Data availability statement

The raw data supporting the conclusions of this article will be made available by the authors on request.

## CRediT authorship contribution statement

**Marie Halfmann:** Writing – original draft, Visualization, Investigation, Formal analysis, Data curation. **Noah Castioni:** Writing – original draft, Validation, Formal analysis, Data curation. **Lea Wetzel:** Writing – review & editing, Software, Methodology, Formal analysis, Data curation, Conceptualization. **Anne Koopmann:** Writing – review & editing, Supervision, Investigation, Conceptualization. **Sarah König:** Validation, Supervision, Resources, Formal analysis. **Astrid Schmieder:** Writing – review & editing, Validation, Supervision, Project administration, Funding acquisition, Formal analysis, Data curation, Conceptualization.

## Declaration of competing interest

The authors declare the following financial interests/personal relationships which may be considered as potential competing interests:Astrid Schmieder reports administrative support, article publishing charges, and equipment, drugs, or supplies were provided by Clinic for Dermatology, Venereology and Allergology, University Hospital Wuerzburg, Germany. Astrid Schmieder reports a relationship with Clinic for Dermatology, Venereology and Allergology, University Hospital Wuerzburg, Germany that includes: employment.
