# Supplementary material for: Effects of the COVID-19 pandemic on the mental health of medical students and young physicians in Germany: Gender-specific results of an online survey
Source: Heliyon. 2023 Dec 19;10(1):e23727. doi: 10.1016/j.heliyon.2023.e23727 (PMC10788433; doi:10.1016/j.heliyon.2023.e23727)
Supplement: Multimedia component 1 [file mmc1.docx]

| *Attachment 1: Pairwise comparisons, post-hoc Dunn-Bonferroni tests; subjective anxiety* | | | | | |
| --- | --- | --- | --- | --- | --- |
| Sample 1-Sample 2 | Test Statistics | Standard Error | Standard Test Statistics | Sig. | Adap. Sig.^a^ |
| Anxiety_Su_2021-Anxiety_Su_2020 | ,507 | ,118 | 4,293 | ,000 | ,000 |
| Anxiety_Su_2021-Anxiety_Sp_2021 | 1,057 | ,118 | 8,941 | ,000 | ,000 |
| Anxiety_Su_2021-Anxiety_A_2020 | 1,434 | ,118 | 12,133 | ,000 | ,000 |
| Anxiety_Su_2021-Anxiety_A_2021 | -1,460 | ,118 | -12,354 | ,000 | ,000 |
| Anxiety_Su_2021- Anxiety _Sp_2020 | 1,996 | ,118 | 16,882 | ,000 | ,000 |
| Anxiety_Su_2021-Anxiety_W_2020 | 2,259 | ,118 | 19,111 | ,000 | ,000 |
| Anxiety_Su_2020-Anxiety_Sp_2021 | -,549 | ,118 | -4,648 | ,000 | ,000 |
| Anxiety_Su_2020-Anxiety_A_2020 | -,927 | ,118 | -7,839 | ,000 | ,000 |
| Anxiety_Su_2020-Anxiety_A_2021 | -,953 | ,118 | -8,061 | ,000 | ,000 |
| Anxiety_Su_2020-Anxiety_Sp_2020 | 1,488 | ,118 | 12,589 | ,000 | ,000 |
| Anxiety_Su_2020-Anxiety_W_2020 | -1,751 | ,118 | -14,818 | ,000 | ,000 |
| Anxiety_Sp_2021-Anxiety_A_2020 | ,377 | ,118 | 3,191 | ,001 | ,030 |
| Anxiety_Sp_2021-Anxiety_A_2021 | -,403 | ,118 | -3,413 | ,001 | ,013 |
| Anxiety_Sp_2021-Anxiety_Sp_2020 | ,939 | ,118 | 7,941 | ,000 | ,000 |
| Anxiety_Sp_2021-Anxiety_W_2020 | 1,202 | ,118 | 10,170 | ,000 | ,000 |
| Anxiety_A_2020-Anxiety_A_2021 | -,026 | ,118 | -,222 | ,825 | 1,000 |
| Anxiety_A_2020-Anxiety_Sp_2020 | ,561 | ,118 | 4,749 | ,000 | ,000 |
| Anxiety_A_2020-Anxiety_W_2020 | -,825 | ,118 | -6,978 | ,000 | ,000 |
| Anxiety_A_2021-Anxiety_Sp_2020 | ,535 | ,118 | 4,528 | ,000 | ,000 |
| Anxiety_A_2021-Anxiety_W_2020 | ,799 | ,118 | 6,757 | ,000 | ,000 |
| Anxiety_Sp_2020-Anxiety_W_2020 | -,263 | ,118 | -2,229 | ,026 | ,542 |

| Each row tests the null hypothesis that the distributions in sample 1 and sample 2 are the same. |
| --- |
| Asymptotic significances (two-sided tests) are shown.   1. The significance level is .050. |
