# Supplementary material for: Effects of the COVID-19 pandemic on the mental health of medical students and young physicians in Germany: Gender-specific results of an online survey
Source: Heliyon. 2023 Dec 19;10(1):e23727. doi: 10.1016/j.heliyon.2023.e23727 (PMC10788433; doi:10.1016/j.heliyon.2023.e23727)
Supplement: Multimedia component 2 [file mmc2.docx]

| *Attachment 2: Pairwise comparisons, post-hoc Dunn-Bonferroni tests; subjective burden* | | | | | |
| --- | --- | --- | --- | --- | --- |
| Sample 1-Sample 2 | Test Statistics | Standard Error | Standard Test Statistics | Sig. | Adap. Sig.^a^ |
| Burden_Su_2020-Burden_Su_2021 | -,135 | ,118 | -1,146 | ,252 | 1,000 |
| Burden_Su_2020-Burden_Sp_2020 | ,946 | ,118 | 8,004 | ,000 | ,000 |
| Burden_Su_2020-Burden_A_2020 | -1,157 | ,118 | -9,790 | ,000 | ,000 |
| Burden_Su_2020-Burden_Sp_2021 | -1,181 | ,118 | -9,992 | ,000 | ,000 |
| Burden_Su_2020-Burden_A_2021 | -1,357 | ,118 | -11,481 | ,000 | ,000 |
| Burden_Su_2020-Burden_W_2020 | -1,935 | ,118 | -16,369 | ,000 | ,000 |
| Burden_Su_2021-Burden_Sp_2020 | ,811 | ,118 | 6,858 | ,000 | ,000 |
| Burden_Su_2021-Burden_A_2020 | 1,022 | ,118 | 8,644 | ,000 | ,000 |
| Burden_Su_2021-Burden_Sp_2021 | 1,046 | ,118 | 8,846 | ,000 | ,000 |
| Burden_Su_2021-Burden_A_2021 | -1,222 | ,118 | -10,334 | ,000 | ,000 |
| Burden_Su_2021-Burden_W_2020 | 1,799 | ,118 | 15,223 | ,000 | ,000 |
| Burden_Sp_2020-Burden_A_2020 | -,211 | ,118 | -1,786 | ,074 | 1,000 |
| Burden_Sp_2020-Burden_Sp_2021 | -,235 | ,118 | -1,988 | ,047 | ,982 |
| Burden_Sp_2020-Burden_A_2021 | -,411 | ,118 | -3,476 | ,001 | ,011 |
| Burden_Sp_2020-Burden_W_2020 | -,989 | ,118 | -8,365 | ,000 | ,000 |
| Burden_A_2020-Burden_Sp_2021 | ,024 | ,118 | ,203 | ,839 | 1,000 |
| Burden_A_2020-Burden_A_2021 | -,200 | ,118 | -1,691 | ,091 | 1,000 |
| Burden_A_2020-Burden_W_2020 | ,778 | ,118 | 6,579 | ,000 | ,000 |
| Burden_Sp_2021-Burden_A_2021 | -,176 | ,118 | -1,488 | ,137 | 1,000 |
| Burden_Sp_2021-Burden_W_2020 | -,754 | ,118 | -6,377 | ,000 | ,000 |
| Burden_A_2021-Burden_W_2020 | ,578 | ,118 | 4,889 | ,000 | ,000 |

| Each row tests the null hypothesis that the distributions in sample 1 and sample 2 are the same. |
| --- |
| Asymptotic significances (two-sided tests) are shown.   1. The significance level is .050. |
