# Supplementary material for: Effects of the COVID-19 pandemic on the mental health of medical students and young physicians in Germany: Gender-specific results of an online survey
Source: Heliyon. 2023 Dec 19;10(1):e23727. doi: 10.1016/j.heliyon.2023.e23727 (PMC10788433; doi:10.1016/j.heliyon.2023.e23727)
Supplement: Multimedia component 3 [file mmc3.docx]

**1. What gender do you assign yourself to?**

Highlight only one circle

- Woman
- Men
- Other:

**2. Age?**

**3. In which years of medical training/medical studies are you currently in?**

Highlight only one circle

- 1st-2nd year
- 3rd year
- 4th-5th year
- 6th year
- Other:

**4. When did you receive your license to practice medicine?**

Highlight only one circle

- I graduated in 2020 or 2021.
- I graduated between 2015 and 2019.
- I graduated between 2010 and 2014.
- I haven't approved yet.

**5. If you are already a doctor, in which specialty do you work?**

**6. Your marital status?**

Highlight only one circle

- Married
- Single/ Living alone
- In a relationship or engaged
- Living with partner
- Living separately
- Divorced
- Widowed
- Other

**7. How would you assess your socioeconomic status?**

Highlight only one circle

- Low
- Insufficient
- Medium
- Sufficient
- High

**8. How satisfied are you with your daily working/study time?**

Highlight only one circle

1-2-3-4-5

**9. How satisfied are you with the working conditions/conditions at your university?**

Highlight only one circle

1-2-3-4-5

**10. Have you worked specifically with COVID-19 patients?**

Highlight only one circle

Yes/no

**11. What kind of work have you done with/on COVID patients? (As a student, volunteer,**

**Employee, diagnostics, treatment, laboratory, research, development, etc.)**

- Volunteer
- Employee
- Diagnostics
- Treatment
- Vaccination
- Laboratory
- Research
- Development
- Not applicable
- Other

**12. Is the equipment in the place where you work/study sufficient to protect yourself from**

**Infectious diseases? (Especially before SARS-CoV-2)**

Highlight only one circle

1-2-3-4-5

**13. Were the protection conditions at your place of study/workplace in the period before the Corona pandemic sufficient?**

Highlight only one circle

1-2-3-4-5

**14. Have you not adequately felt protected at any time during the Corona pandemic**

**(protective equipment, safety precautions, etc.) at the place of study/workplace?**

Highlight only one circle

Yes/no/I don´t know

**15. Are you satisfied with the attitude (respect, recognition, appreciation, etc.) of society towards Healthcare professionals?**

Highlight only one circle

1-2-3-4-5

**16. Do you think the way politicians deal with health professionals is appropriate?**

Highlight only one circle

1-2-3-4-5

**17. Have you ever been (in your capacity as a healthcare professional) exposed to violence of patients or relatives in the health care facility (hospital, university hospital, etc.)?**

Select answers that apply

- Verbal attack

1. I didn't experience it
2. I experienced it
3. Iexperienced it (e.g.with colleagues)

- Physical attack

1. I didn't experience it
2. I experienced it
3. Iexperienced it (e.g.with colleagues)

- Sexual assault
  1. I didn't experience it
  2. I experienced it
  3. **Iexperienced it (e.g.with colleagues)**

**18. Have you ever been (in the capacity of a health professional) exposed to violence in the field of health from your colleagues, supervisors or fellow students?**

Select answers that apply

- Verbal attack

1. I didn't experience it
2. I experienced it
3. Iexperienced it (e.g.with colleagues)

- Physical attack

1. I didn't experience it
2. I experienced it
3. Iexperienced it (e.g.with colleagues)

- Sexual assault
  1. I didn't experience it
  2. I experienced it
  3. Iexperienced it (e.g.with colleagues)

**19. Have you felt discriminated at any time in your medical studies (for any reason)?**

Highlight only one circle

1-2-3-4-5

**20. How do you assess your personally perceived fear/insecurity over the course of the pandemic?**

Select only one circle per row

- Spring 2020
  - None at all fear/insecurity
  - Sometimes fear/insecurity
  - Strong fear/insecurity
- Summer 2020
  - None at all fear/insecurity
  - Sometimes fear/insecurity
  - Strong fear/insecurity
- Autumn 2020
  - None at all fear/insecurity
  - Sometimes fear/insecurity
  - Strong fear/insecurity
- Winter 2020
  - None at all fear/insecurity
  - Sometimes fear/insecurity
  - Strong fear/insecurity
- Spring 2021
  - None at all fear/insecurity
  - Sometimes fear/insecurity
  - Strong fear/insecurity
- Summer 2021
  - None at all fear/insecurity
  - Sometimes fear/insecurity
  - Strong fear/insecurity
- Autumn 2021
  - None at all fear/insecurity
  - Sometimes fear/insecurity
  - Strong fear/insecurity

**21. Do you feel that your education and training has been affected during the Corona pandemic?**

Highlight only one circle

Not at all/a little bit/sometimes/strongly

**22. Were you diagnosed with a mental illness before the pandemic?**

Mark only one circle

Yes/no

**23. If your answer to the previous question "Yes", would you like to specify the diagnosis (ICD-10 code)?**

You do not have to answer this question.

**24. Have you had any mental illnesses during the Corona pandemic?**

Highlight only one circle

Yes/no

**25. Have you had some form of psychosocial support?**

**Have you received support? For example, from institutions such as student organizations, trade unions, Self-help programs, etc.**

- Student Organizations
- Unions
- Self-help programs
- Supervision
- Psychotherapy
- None
- Other

**26. In your opinion, how great is the overall threat (health, economic, etc.) in the coming months?**

Select only one circle per row

- For me privately (low/medium/great)
- for Germany (low/medium/great)
- worldwide (low/medium/great)

**27. How were the following aspects of your life affected by the pandemic in the spring of 2020?**

Select only one circle per row

- Family life (such as spouse, children, parents) (positive/negative/neutral)
- Friendship and social relationships (positive/negative/neutral)
- Work and professional development (positive/negative/neutral)

**28. How were the following aspects of your life affected by the pandemic in the summer of 2020?**

Select only one circle per row

- Family life (such as spouse, children, parents) (positive/negative/neutral)
- Friendship and social relationships (positive/negative/neutral)
- Work and professional development (positive/negative/neutral)

**29. How were the following aspects of your life affected by the pandemic in the autumn of 2020?**

Select only one circle per row

- Family life (such as spouse, children, parents) (positive/negative/neutral)
- Friendship and social relationships (positive/negative/neutral)
- Work and professional development (positive/negative/neutral)

**30. How were the following aspects of your life affected by the pandemic in the winter of 2020?**

Select only one circle per row

- Family life (such as spouse, children, parents) (positive/negative/neutral)
- Friendship and social relationships (positive/negative/neutral)
- Work and professional development (positive/negative/neutral)

**31. How were the following aspects of your life affected by the pandemic in the spring of 2021?**

Select only one circle per row

- Family life (such as spouse, children, parents) (positive/negative/neutral)
- Friendship and social relationships (positive/negative/neutral)
- Work and professional development (positive/negative/neutral)

**32. How were the following aspects of your life affected by the pandemic in the summer of 2021?**

Select only one circle per row

- Family life (such as spouse, children, parents) (positive/negative/neutral)
- Friendship and social relationships (positive/negative/neutral)
- Work and professional development (positive/negative/neutral)

**33. How were the following aspects of your life affected by the pandemic in the autumn of 2021?**

Select only one circle per row

- Family life (such as spouse, children, parents) (positive/negative/neutral)
- Friendship and social relationships (positive/negative/neutral)
- Work and professional development (positive/negative/neutral)

**34. Evaluate your personal burden during this process.**

Select only one circle per row

- Spring 2020
  - None
  - Less than usual
  - constant
  - More than usual
  - Very strong
- Summer 2020
  - None
  - Less than usual
  - constant
  - More than usual
  - Very strong
- Autumn 2020
  - None
  - Less than usual
  - constant
  - More than usual
  - Very strong
- Winter 2020
  - None
  - Less than usual
  - constant
  - More than usual
  - Very strong
- Spring 2021
  - None
  - Less than usual
  - constant
  - More than usual
  - Very strong
- Summer 2021
  - None
  - Less than usual
  - constant
  - More than usual
  - Very strong
- Autumn 2021
  - None
  - Less than usual
  - constant
  - More than usual
  - Very strong

**35. During the course of the Corona pandemic, was there an event in the hospital that had a positive or negative impact on you as a physician? In your role as a physician?**

**36. I feel tense or overstimulated.**

Select only one circle per row

- Before the outbreak of the pandemic (Hardly or not at all/ Only a little bit/ often/Very much)
- After the outbreak of the pandemic (Hardly or not at all/ Only a little bit/ often/Very much)

**37. I can still rejoice today as I did before**

Select only one circle per row

- Before the outbreak of the pandemic (not at all/ only a little bit/ not so much/exactly as before)
- After the outbreak of the pandemic (not at all/ only a little bit/ not so much/exactly as before)

**38. I am overcome by an anxious premonition that something terrible could happen.**

Select only one circle per row

- Before the outbreak of the pandemic (not at all/ only a little bit/ not so much/yes, strongly)
- After the outbreak of the pandemic (not at all/ only a little bit/ not so much/yes, strongly)

**39. I sometimes have an uneasy gut feeling.**

Select only one circle per row

- Before the outbreak of the pandemic (Hardly or not at all/ Only a little bit/ often/Very much)
- After the outbreak of the pandemic (Hardly or not at all/ Only a little bit/ often/Very much)

**40. I feel slowed down in my activities.**

Select only one circle per row

- Before the outbreak of the pandemic (Hardly or not at all/ Only a little bit/ often/Very much)
- After the outbreak of the pandemic (Hardly or not at all/ Only a little bit/ often/Very much)

**41. I have lost interest in my outward appearance.**

Select only one circle per row

- Before the outbreak of the pandemic (Hardly or not at all/ Only a little bit/ often/Very much)
- After the outbreak of the pandemic (Hardly or not at all/ Only a little bit/ often/Very much)

**42. I can laugh and see the funny side of things.**

Select only one circle per row

- Before the outbreak of the pandemic (not at all/ less than before/ not so much/exactly as before)
- After the outbreak of the pandemic (not at all/ less than before / not so much/exactly as before)

**43. I have disturbing thoughts going through my head.**

Select only one circle per row

- Before the outbreak of the pandemic (A large part of the time/relatively often/from time to time, but not too often/only occasionally, never)
- After the outbreak of the pandemic (A large part of the time/relatively often/from time to time, but not too often/only occasionally, never)

**44. I feel lucky.**

Select only one circle per row

- Before the outbreak of the pandemic (A large part of the time/relatively often/from time to time, but not too often/only occasionally, never)
- After the outbreak of the pandemic (A large part of the time/relatively often/from time to time, but not too often/only occasionally, never)

**45. I can sit quietly and relax.**

Select only one circle per row

- Before the outbreak of the pandemic (A large part of the time/relatively often/from time to time, but not too often/only occasionally, never)
- After the outbreak of the pandemic (A large part of the time/relatively often/from time to time, but not too often/only occasionally, never)

**46. I feel restless, I always have to be on the move.**

Select only one circle per row

- Before the outbreak of the pandemic (A large part of the time/relatively often/from time to time, but not too often/only occasionally, never)
- After the outbreak of the pandemic (A large part of the time/relatively often/from time to time, but not too often/only occasionally, never)

**47. I look to the future with joy.**

Select only one circle per row

- Before the outbreak of the pandemic (A large part of the time/relatively often/from time to time, but not too often/only occasionally, never)
- After the outbreak of the pandemic (A large part of the time/relatively often/from time to time, but not too often/only occasionally, never)

**48. I am suddenly overcome by a panic-like state.**

Select only one circle per row

- Before the outbreak of the pandemic (A large part of the time/relatively often/from time to time, but not too often/only occasionally, never)
- After the outbreak of the pandemic (A large part of the time/relatively often/from time to time, but not too often/only occasionally, never)

**49. I can enjoy a good book, radio or television show.**

Select only one circle per row

- Before the outbreak of the pandemic (A large part of the time/relatively often/from time to time, but not too often/only occasionally, never)
- After the outbreak of the pandemic (A large part of the time/relatively often/from time to time, but not too often/only occasionally, never)

**50. Do you suffer from insomnia because of your worries?**

Select only one circle per row

- Before the outbreak of the pandemic (A large part of the time/relatively often/from time to time, but not too often/only occasionally, never)
- After the outbreak of the pandemic (A large part of the time/relatively often/from time to time, but not too often/only occasionally, never)

**51. Do you feel like you're living in constant trouble?**

Highlight only one circle per row

- Before the outbreak of the pandemic (A large part of the time/relatively often/from time to time, but not too often/only occasionally, never)
- After the outbreak of the pandemic (A large part of the time/relatively often/from time to time, but not too often/only occasionally, never)

**52. Are you able to focus sufficiently on your work?**

Select only one circle per row

- Before the outbreak of the pandemic (A large part of the time/relatively often/from time to time, but not too often/only occasionally, never)
- After the outbreak of the pandemic (A large part of the time/relatively often/from time to time, but not too often/only occasionally, never)

**53. Do you feel that you can positively influence your environment?**

Select only one circle per row

- Before the outbreak of the pandemic (A large part of the time/relatively often/from time to time, but not too often/only occasionally, never)
- After the outbreak of the pandemic (A large part of the time/relatively often/from time to time, but not too often/only occasionally, never)

**54. Do you think that you are able to solve your problems yourself?**

Select only one circle per row

- Before the outbreak of the pandemic (A large part of the time/relatively often/from time to time, but not too often/only occasionally, never)
- After the outbreak of the pandemic (A large part of the time/relatively often/from time to time, but not too often/only occasionally, never)

**55. Do you have trouble making decisions?**

Select only one circle per row

- Before the outbreak of the pandemic (A large part of the time/relatively often/from time to time, but not too often/only occasionally, never)
- After the outbreak of the pandemic (A large part of the time/relatively often/from time to time, but not too often/only occasionally, never)

**56. Do you feel that the difficulties you face are insurmountable?**

Select only one circle per row

- Before the outbreak of the pandemic (A large part of the time/relatively often/from time to time, but not too often/only occasionally, never)
- After the outbreak of the pandemic (A large part of the time/relatively often/from time to time, but not too often/only occasionally, never)

**57. Do you feel happy when you reflect on what is happening from different angles?**

Highlight only one circle per row

- Before the outbreak of the pandemic (A large part of the time/relatively often/from time to time, but not too often/only occasionally, never)
- After the outbreak of the pandemic (A large part of the time/relatively often/from time to time, but not too often/only occasionally, never)

**58. Can you enjoy your daily work?**

Select only one circle per row

- Before the outbreak of the pandemic (A large part of the time/relatively often/from time to time, but not too often/only occasionally, never)
- After the outbreak of the pandemic (A large part of the time/relatively often/from time to time, but not too often/only occasionally, never)

**59. Do you feel unhappy and depressed?**

Select only one circle per row

- Before the outbreak of the pandemic (A large part of the time/relatively often/from time to time, but not too often/only occasionally, never)
- After the outbreak of the pandemic (A large part of the time/relatively often/from time to time, but not too often/only occasionally, never)

**60. Do you agree with the statement "I have low self-confidence"?**

Select only one circle per row

- Before the outbreak of the pandemic (A large part of the time/relatively often/from time to time, but not too often/only occasionally, never)
- After the outbreak of the pandemic (A large part of the time/relatively often/from time to time, but not too often/only occasionally, never)

**61. Do you agree with the statement "I have low self-esteem"?**

Select only one circle per row

- Before the outbreak of the pandemic (A large part of the time/relatively often/from time to time, but not too often/only occasionally, never)
- After the outbreak of the pandemic (A large part of the time/relatively often/from time to time, but not too often/only occasionally, never)

**62. To what extent did your work/study activity influence your answers in the second part of the survey?**

Highlight only one circle

- I am very relaxed/balanced when I work/study. It is an absolutely positive influencing factor.
- I'm a bit more relaxed/balanced than usual when I'm working/studying. Maybe it's a minor
- positive influencing factor.
- It didn't affect my answers.
- I'm a little more restless and unhappy than usual when I'm working/studying. Maybe it's a minor negative influencing factor.
- I am very restless and unhappy when I work/study. I feel exhausted. It's an absolutely

negative influencing factor.

**63. Have you ever had to perform a task in your everyday work or as part of your studies,**

**whose solution has exceeded your personal competence/knowledge/skills? Were you**

**forced to do it (e.g. by superiors, patients, etc.)?**

Highlight only one circle

Yes/no

**64. If you answered 'yes' to the previous question, please describe, if possible, the situation in question.**

**65. What is your highest school leaving certificate?**

Highlight only one circle

- Abitur
- University
- Postgraduate (Dr.)

**66. Are you currently ill?**

Highlight only one circle

Yes/no

**67. How would you describe your quality of life (in the last two weeks)?**

1-2-3-4-5

**68. How satisfied are you with your current health?**

1-2-3-4-5

**69. Please assess how strongly you have experienced certain things during the past two weeks.**

Select only one circle per row

- How much pain prevents you from doing necessary things? (not at all/a little/mediocre/quite/extreme)
- How much are you dependent on medical treatment, to cope with daily life? (not at all/a little/mediocre/quite/extreme)
- How well can you enjoy your life? (not at all/a little/mediocre/quite/extreme)
- Do you consider your life to be meaningful? (not at all/a little/mediocre/quite/extreme)
- How well can you concentrate? (not at all/a little/mediocre/quite/extreme)
- How safe do you feel in your daily life? (not at all/a little/mediocre/quite/extreme)
- How healthy are the environmental conditions in your country? Residential area? (not at all/a little/mediocre/quite/extreme)

**70. Assess the extent to which you have experienced certain things during the past two weeks**

**or have been able to do certain things.**

Select only one circle per row

- Do you have enough energy for daily life? (Not at all/rather not /ok/ mostly/ completely)
- Can you accept your appearance? (Not at all/rather not /ok/ mostly/ completely)
- Do you have enough money to meet your needs? (Not at all/rather not /ok/ mostly/ completely)
- Do you have access to the information you need for the daily life? (Not at all/rather not /ok/ mostly/ completely)
- Do you have enough opportunities to perfomr leisure activities? (Not at all/rather not /ok/ mostly/ completely)
- How well can you get around? (Not at all/rather not /ok/ mostly/ completely)

**71. Assess how satisfied, happy or well you feel during the past two weeks regarding different aspects of your life.**

Select only one circle per row

How satisfied are you with your sleep? (Very much dissatisfied/dissatisfied/neither/satisfied/Very much satisfied)

How satisfied are you with your ability to do everyday things? (Very much dissatisfied/dissatisfied/neither/satisfied/Very much satisfied)

How satisfied are you with your ability to work? (Very much dissatisfied/dissatisfied/neither/satisfied/Very much satisfied)

How satisfied are you with yourself? (Very much dissatisfied/dissatisfied/neither/satisfied/Very much satisfied)

How satisfied are you with your personal relationships? (Very much dissatisfied/dissatisfied/neither/satisfied/Very much satisfied)

How satisfied are you with your sex life? (Very much dissatisfied/dissatisfied/neither/satisfied/Very much satisfied)

How satisfied are you with the support from your friends? (Very much dissatisfied/dissatisfied/neither/satisfied/Very much satisfied)

How satisfied are you with your living conditions? (Very much dissatisfied/dissatisfied/neither/satisfied/Very much satisfied)

How satisfied are you with your options, to be able to use health services? (Very much dissatisfied/dissatisfied/neither/satisfied/Very much satisfied)

How satisfied are you with the means of transport that are available to you? (Very much dissatisfied/dissatisfied/neither/satisfied/Very much satisfied)

**72. How many times in the past two weeks have you experienced negative feelings such as fear, sadness or depression?**

Highlight only one circle

(never/not often/sometimes/often/always)
